# The Effect of Seasonal Variations in Airborne Particulate Matter on Asthma-Related Airway Inflammation in Mice

**DOI:** 10.3390/ijerph13060579

**Published:** 2016-06-09

**Authors:** Jun Kurai, Masanari Watanabe, Hiroyuki Sano, Degejirihu Hantan, Eiji Shimizu

**Affiliations:** 1 Department of Respiratory Medicine and Rheumatology, Faculty of Medicine, Tottori University, 36-1 Nishi-cho, Yonago 683-8504, Japan; junkurajun@gmail.com (J.K.); degujirefu@med.tottori-u.ac.jp (D.H.); eiji@med.tottori-u.ac.jp (E.S.); 2 Department of Respiratory Medicine and Allergology, Faculty of Medicine, Kinki University, 377-2 Ohnohigashi, Osakasayama 589-0014, Japan; hsano@med.kindai.ac.jp

**Keywords:** airway inflammation, asthma, seasonal variation, NC/Nga mouse model

## Abstract

This study aimed to investigate the effects of winter and spring particulate matter (PM) on airway inflammation and allergies in a mouse asthma model. PM was collected during 7–28 February 2013 (winter) and during 7–28 April 2013 (spring) in Yonago, Japan. NC/Nga mice were co-sensitized using intranasal instillation of the PMs and *Dermatophagoides farinae* (Df) for 5 consecutive days, and were subsequently challenged using intranasal Df at 7 days after the last sensitization. At 24 h after the challenge, serum immunoglobulin levels, differential leukocyte counts, and inflammatory cytokines levels were measured in the mice’s bronchoalveolar lavage fluid (BALF). Compared to co-sensitization using spring PM and Df, winter PM and Df induced greater increases in the BALF neutrophil and eosinophil counts and total serum IgE and IgG2a levels. Furthermore, winter PM-sensitized mice exhibited higher BALF levels of interleukin-5, interleukin-13, interleukin-6, and keratinocyte-derived chemokine. Therefore, we observed seasonal variations in the effects of PM on asthma-related airway inflammation. These findings suggest that the compositions of PM vary according to season, and that it is important to evaluate PM compositions in order to understand the associations between asthma and PM.

## 1. Introduction

Air pollution, including particulate matter (PM), is now the third leading global cause of disability-adjusted life years that are associated with health disorders [1]. Several studies have demonstrated that PM exposure is associated with exacerbated asthma and increased primary care visits, respiratory symptoms, cardiovascular mortality, and lung cancer [2,3,4,5,6,7,8]. Furthermore, other studies have demonstrated that PM is associated with respiratory diseases [9,10] and cardiopulmonary mortality [11]. However, other studies have reported conflicting findings [12,13], and results regarding the relationship between airborne PM and asthma are inconsistent. Moreover, no study has performed a comprehensive quantitative evaluation of PM’s effect on asthma [14], and the reported effects of PM on health disorders, including asthma, are also inconsistent.

Airborne PM is usually categorized based on median aerodynamic particle diameters of <10 μm (PM_10_) and <2.5 μm (PM_2.5_). Thus, previous studies have primarily focused on PM_10_ and PM_2.5_ levels to estimate the associations between airborne PM and health disorders. Airborne PM usually consists of various components from different sources, such as crystal materials and materials from traffic, biomass combustion, waste incineration, industrial processes, transported air pollution, road abrasion and resuspension, car brake debris, and bacterial and fungal dust [15]. In this context, several recent studies have reported that the effects of airborne PM on respiratory diseases vary according to each report [16,17]. Other studies have also demonstrated that airborne PM has city- and season-specific inflammatory potentials [18,19,20,21]. Thus, the toxic effects of airborne PM may vary according to its composition [22].

Airborne PM increases airway inflammation and has immune adjuvant effects in the ovalbumin (OVA)-induced asthma mouse model [23,24,25], which indicates that substances in airborne PM may provoke asthma exacerbation. However, the OVA-induced asthma mouse model uses aluminum as an adjuvant, and airborne PM contains aluminum [23]. In this context, an innovative asthma mouse model that was developed by Shibamori *et al*. exhibits allergic asthma-like reactions after intranasal sensitization using *Dermatophagoides farinae* (Df) [26]. Therefore, this model may be more useful for evaluating the effects of PM on asthma, as the Df-induced asthma model does not require an adjuvant, unlike the OVA-induced model.

The increased levels of airborne PM in China is a serious environmental problem [27,28], and the long-range transportation of aerosols from East Asia to Japan has increased the PM levels in Japan [29,30,31]. Furthermore, the increased use of heating fuel during January and February can worsen the air quality in China. Therefore, we hypothesized that the inflammatory effects of airborne PM on asthma may vary according to season in western Japan, based on the long-range transportation of air pollutants. In this study, we evaluated airway inflammation and immune adjuvant effect in a Df-induced asthma model, where we co-sensitized the mice using Df and winter or spring PM, in order to evaluate the seasonal influences of short-term PM exposure.

## 2. Materials and Methods

### 2.1. Animals

Specific pathogen-free 7-week-old male NC/Nga mice were purchased from Japan SLC Inc. (Hamamatsu, Japan) and acclimatized for 7 days before starting the study. Animals were kept in a vivarium at a constant temperature of 22 °C and were illuminated in 12-h light/dark cycles. Animals were fed standard animal chow daily and had ad libitum access to drinking water. The experimental protocols were approved by the Institutional Animal Care and Use Committee, Faculty of Medicine, Tottori University (12-Y-55).

### 2.2. PM Preparation

Winter PM (PM_1_) was collected in the city of Tottori, Japan during 7–28 February 2013, and spring PM (PM_2_) was collected during 7–28 April 2013. The PM collections were performed using a high-volume air sampler (HV-1000R; Shibata Co., Tokyo, Japan) that was fixed to a building’s roof. The PM was separated according to aerodynamic diameter using five filters (<1.1 µm, 1.1–2.0 µm, 2.0–3.3 µm, 3.3–7.0 µm, and >7.0 μm), and each filter was weighed before and after the sampling (post-sampling weight was obtained after drying the filters in a desiccator). We used PM with a diameter of 3.3–7.0 μm, which contains common ambient urban PM that exhibits a peak at an approximately median aerodynamic diameter of 5 μm [32]. The PM was sterilized at 121 °C for 30 min in an autoclave (Tomy SX-300; Tomy Co., Tokyo, Japan) and stored in a freezer at −20 °C to prevent the growth of bacteria and fungi. The PM was subsequently suspended in normal saline (NS) before being administered to the mice.

### 2.3. Experimental Protocol

After a 7-day acclimatization period, the NC/Nga mice were randomly divided into six groups (*n* = 8 per group) and sensitized to Df (Greer Laboratories Inc., Lenoir, NC, USA), as previously described [26]. For sensitization, the mice were anesthetized using isoflurane inhalation and we performed intranasal installations of a crude Df extract (50 μg in 25 μL of NS) for 5 consecutive days (days 0–4). The Df-sensitized mice were then intranasally challenged using Df at 7 days after the last Df sensitization (day 11) and were subsequently sacrificed at 24 h after the Df challenge. The control group received NS without the Df extract.

To observe the immune adjuvant effects and airway inflammation that was induced by the PM, mice were co-sensitized using intranasal instillations of NS or PM (0.1 mg in 25 μL of NS) and Df for 5 consecutive days (days 0–4). The three groups were: (i) co-sensitization using NS and Df with a Df challenge (NS + Df/Df mice); (ii) co-sensitization using PM_1_ and Df with a Df challenge (PM_1_ + Df/Df mice); and (iii) co-sensitization using PM_2_ and Df with a Df challenge (PM_2_ + Df/Df mice). The one-time PM instillation dose (0.1 mg/mouse) was conducted according to a previous report [25,33].

### 2.4. Bronchoalveolar Lavage

After the mice were anesthetized using isoflurane, their tracheas were cannulated. The BALF was obtained after five installations of NS (1.0 mL) into the lungs, with gentle handling to maximize the BALF recovery. The BALF from each mouse was centrifuged at 300× *g* for 5 min at 4 °C. The cell pellet was used for the cell counting, and the supernatant was used for the cytokine analyses. The cells were diluted in Turk’s fluid and a total count was obtained using a hemocytometer. The differential leukocyte counts were obtained using microscopic evaluations and quantitative analyses of methanol-fixed cytospin preparations that were stained using Diff Quick (Fisher Scientific, Pittsburgh, PA, USA).

### 2.5. Enzyme-Linked Immunosorbent Assays for Serum Total Immunoglobulin

Serum total IgE and IgG2a levels were measured using OptEIA Mouse kits (BD Pharmingen, San Diego, CA, USA), according to the manufacturer’s instructions. The assays were quantified based on optical density (450 nm), which was read using a calibrated Sunrise microplate reader (Tecan Group, Kawasaki, Japan) and XFluor4 software (Tecan Group).

### 2.6. Quantitative Evaluation of Cytokine and Chemokine Levels

We evaluated the BALF levels of interferon (IFN)-γ, interleukin (IL)-13, IL-5, IL-6, keratinocyte-derived chemokine (KC/CXCL1), and macrophage inflammatory protein (MIP)-2 (KC/CXCL1 and MIP-2/CXCL2 are murine homologues of human IL-8) using the appropriate enzyme immunoassay (EIA) kits (R&D Systems Europe, Abingdon, UK). The BALF was not diluted for the IFN-γ assay, and was diluted to 1:5 for the IL-13, IL-5, IL-6, KC/CXCL1, and MIP-2/CXCL2 assays. All EIAs were performed according to the manufacturer’s instructions.

### 2.7. Endotoxin Analysis

Levels of endotoxin were evaluated using the ToxinSensor Chromogenic LAL endotoxin assay (GenScript, Piscataway, NJ, USA), according to the manufacturer’s instructions. This assay has a sensitivity range of 0.01–1 EU/mL. The pH of the PM was measured using a MP220 meter (Mettler Toledo, Schwerzenbach, Switzerland).

### 2.8. Statistical Analysis

Data were expressed as mean ± standard deviation. Inter-group comparisons were performed using one-way analysis of variance. All analyses were performed using GraphPad Prism software (version 5.02; GraphPad Software, San Diego, CA, USA), and differences were considered statistically significant at a *p*-value of <0.05.

## 3. Results

### 3.1. Co-Sensitization Using PM_*1*_ and Df Induced the Greatest Increase in Airway Inflammation

**Figure 1:**
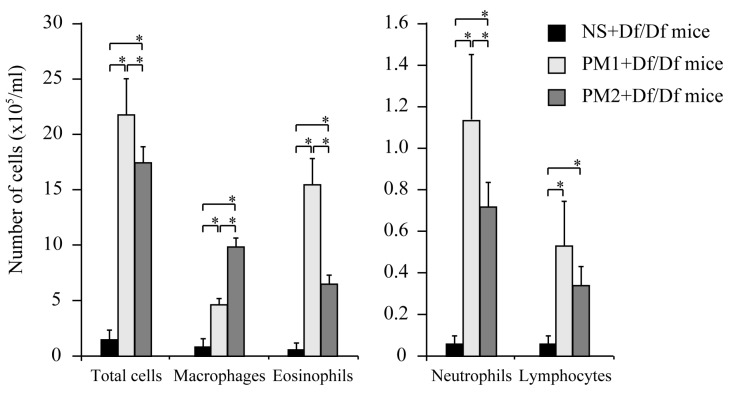
Total and differential leukocyte counts in bronchoalveolar lavage fluid (BALF). Mice were divided to three sensitized groups that were all subsequently challenged using *Dermatophagoides farina* (Df): normal saline with Df (NS + Df/Df), particulate matter 1 (7–28 February 2013) with Df (PM_1_ + Df/Df), and particulate matter 2 (7–28 April 2013) with Df (PM_2_ + Df/Df). Cell counts (eosinophils, macrophages, neutrophils, and lymphocytes) in the BALF were obtained at 24 h after the challenge on day 11. The total cell counts from PM_1_ + Df/Df mice were significantly higher, compared to those from PM_2_ + Df/Df mice. Data for each group are expressed as mean ± standard deviation, and the results are from eight mice per group. * *p* < 0.05.

The PM_1_ + Df/Df mice and PM_2_ + Df/Df mice exhibited significant increases in their total BALF cell counts, compared to the NS + Df/Df mice (*p* < 0.05). Compared to the differential counts from the control mice, the greatest increase was observed in the neutrophil counts. In addition, compared to the PM_2_ + Df/Df mice, the PM_1_ + Df/Df mice exhibited a greater increase in the total cell count (1.3-fold increase *vs*. PM_2_ + Df/Df), which was related to increases in the neutrophil count (1.6-fold) and eosinophil count (2.4-fold) (Figure 1). These data indicate that PM_1_ induced the greatest recruitment of inflammatory cells into the lung tissues.

### 3.2. Co-Sensitization Using PM_*1*_ and Df Induced the Greatest Production of Th2 and Inflammatory Cytokines

**Figure 2:**
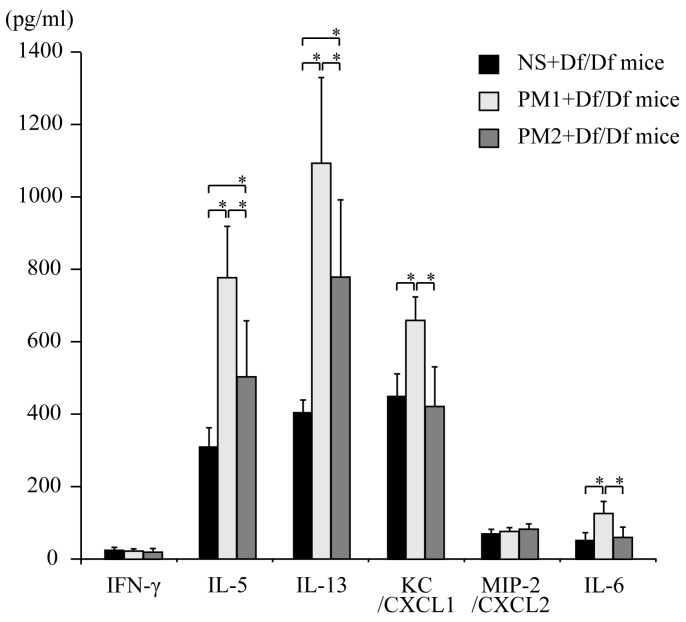
Cytokine and chemokine levels in bronchoalveolar lavage fluid (BALF). The BALF cytokine and chemokine expression profiles were analyzed using enzyme immunoassays for interferon-γ, interleukin-5, interleukin-13, keratinocyte-derived chemokine, macrophage inflammatory protein-2, and interleukin-6. Data for each group are expressed as mean ± standard deviation, and the results are from six mice per group. * *p* < 0.05.

We measured BALF cytokine levels to investigate the mechanisms through which PM caused an allergic airway response in our Df-induced asthma model. Similar to the PM-induced inflammatory cell recruitment, PM induced the production of several important asthma-related cytokines. Compared to the NS + Df/Df mice, the PM_2_ + Df/Df mice exhibited higher BALF levels of Th2 cytokines (IL-5 and IL-13). Moreover, compared to the NS + Df/Df mice and the PM_2_ + Df/Df mice, the PM_1_ + Df/Df mice exhibited higher BALF levels of Th2 cytokines (IL-5 and IL-13) and inflammatory cytokines (KC and IL-6), with the exception of MIP-2 (a neutrophil activation chemokine) (Figure 2). The administration of PM_1_ and PM_2_ did not induce an increase in the BALF levels of IFN-γ (a Th1 cytokine).

### 3.3. Co-Sensitization Using PM_*1*_ and Df Provided the Strongest Immune Adjuvant Effects

**Figure 3:**
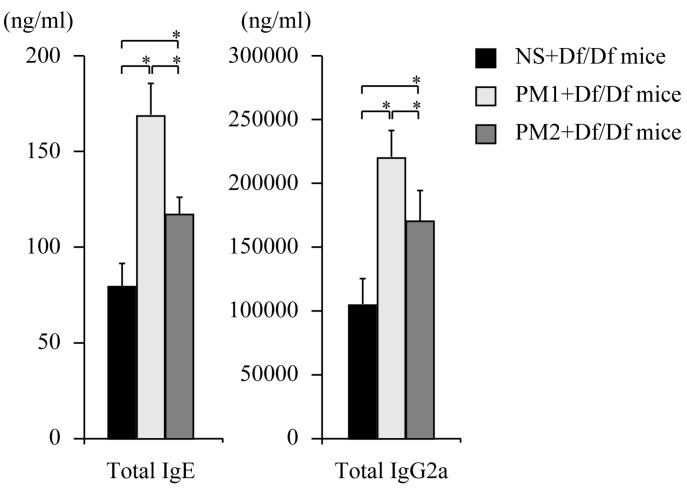
Total IgE and total IgG2a levels in serum. The levels of serum total IgE and total IgG2a were detected using enzyme immunoassays and presented as serum concentrations. Data for each group are expressed as mean ± standard deviation, and the results are from six mice per group. * *p* < 0.05.

Serum immunoglobulin levels were evaluated on day 12 to examine the immune adjuvant effects of PM in Df-sensitized mice. Compared to the NS + Df/Df mice, the PM_1_ + Df/Df mice and PM_2_ + Df/Df mice exhibited significantly increased serum total IgE levels (2.1-fold and 1.5-fold, respectively, as immunoglobulin concentrations, *p* < 0.05) and serum total IgG2a levels (1.9-fold and 1.4-fold, respectively, as immunoglobulin concentrations, *p* < 0.05) (Figure 3). Furthermore, the PM_1_ + Df/Df mice exhibited a greater increase in the total IgE and IgG2a levels, compared to the PM_2_ + Df/Df mice. These data indicate that PM_1_ provided the most potent immune adjuvant effects.

### 3.4. Endotoxin Concentrations and pH Values in the PM

At a PM concentration of 1 mg/mL, the endotoxin levels were 1.10 EU/mL in PM_1_ and in 0.89 EU/mL in PM_2_. At the same PM concentration, the pH values were 7.8 in PM_1_ and 7.7 in PM_2_.

## 4. Discussion

These findings indicate that PM from different seasons (winter and spring) in western Japan had significantly different immune adjuvant effects and effects on airway inflammation in a mouse model of asthma. For example, compared to the spring PM, the winter PM significantly increased the number of eosinophils and neutrophils in the BALF, and significantly augmented the airway inflammation. The winter PM also induced the greatest increases in serum total IgE and IgG2a levels, and the production of Th2 cytokines (IL-5 and IL-13) and inflammatory cytokines (KC and IL-6). These results suggest that winter PM from western Japan has greater potential for allergic toxicity, compared to spring PM.

It has been hypothesized that the seasonal toxicity of PM exposure might be linked to differences in PM composition and variations in the proportional size distributions [34,35]. A previous study found that PM from Italy that was collected during different seasons (summer and winter) caused different acute toxic effects in a mouse model [36]. However, another researcher did not find any seasonal effects or significant interactions between season and particle size or between sampling location and *in vitro* exposure [37]. These discrepancies may be related to differences in the composition of the PM.

Inhaled endotoxin is associated with neutrophilic airway inflammation in patients with asthma and healthy individuals [38,39]. Endotoxin in environmental aerosols can also augment airway inflammation in a murine model of asthma [40]. Thus, endotoxin plays an important role in airborne PM-induced airway inflammation. In the present study, the winter PM exhibited higher endotoxin levels, compared to the spring PM, and endotoxin may be an important factor that partially explains the seasonal effects that we observed. In addition, KC and IL-6 may be key inflammatory mediators for the increase in airway inflammation. In contrast, MIP–2 and IFN-γ had no effect on airway inflammation that was augmented using airborne PM.

Kumar *et al*. [21] compared the production of pro-inflammatory cytokines after exposure to various PM sources, and suggested that the iron content of airborne PM might be an important mediator of airway epithelial injury. A recent *in vivo* study also revealed an association between the iron content of inhaled particulates and pulmonary function deficits [41]. Thus, in addition to endotoxin in environmental aerosols, metal components (e.g., iron) may also play important roles in PM-induced airway inflammation. However, we were unable to analyze the specific compositions of our PM samples, based on the small amounts that we collected. Therefore, we were unable to identify the component(s) that may play important roles in induced airway inflammation, and further studies are needed to identify the component(s) that are responsible for the seasonal variations in PM-induced airway inflammation.

In January 2013, high concentrations of air pollution and PM were identified in various Chinese cities [42]. Furthermore, Yamazaki *et al*. found that the mean monthly PM concentrations in western Japan during January–March 2013 (winter) were slightly higher than the concentrations during the winters of 2011 or 2012 [2]. They also found that ozone levels may be associated with primary care visits at night for asthma attacks, although there was no association between the daily mean concentrations of fine PM and primary care visits [2]. Nevertheless, our findings that winter PM from the same period had greater allergic toxicity, compared to spring PM, may support their epidemiological findings.

February is a boundary between winter and spring in East Asia, which allowed us to compare the seasonal variations in East Asian air pollution, [43,44]. In East Asia, PM_10_ levels change in March and are maintained near their highest levels from March to April [45]. A high atmospheric level of Japanese cedar pollen is also present during March in Japan [46]. Therefore, the present study defined winter PM as PM that was collected in February and spring PM as PM that was collected in April.

Inhaled airborne PM can affect different parts of the respiratory tract according to particulate size [47]. Coarse PM (PM_2.5–10_) is primarily deposited in the bronchus, and fine PM (PM_2.5_) is more likely to be deposited deeper in the alveolar regions. As we aimed to investigate the seasonal differences in the inflammatory effects of airborne PM on asthma, we used PM with a diameter of 3.3–7.0 µm.

## 5. Conclusions

We conclude that winter PM from western Japan had greater immune adjuvant effects and increased neutrophilic airway inflammation, compared to spring PM. This relationship may be mediated by endotoxins that are attached to airborne particles. Therefore, further studies are needed to identify the substances that are responsible for the seasonal differences in the effects of PM.

## Abbreviations

The following abbreviations are used in this manuscript:

BALF	bronchoalveolar lavage fluid
Df	*Dermatophagoides farina*
EIA	enzyme immunoassay
IFN	interferon
IL	interleukin
KC	keratinocyte-derived chemokine
MIP	macrophage inflammatory protein
NS	normal saline
PM	particulate matter
PM_1_	particulate matter from 7–28 February 2013
PM_2_	particulate matter from 7–28 April 2013

## References

[1] Lim S.S., Vos T., Flaxman A.D., Danaei G., Shibuya K., Adair-Rohani H., Amann M., Anderson H.R., Andrews K.G., Aryee M. (2012). A comparative risk assessment of burden of disease and injury attributable to 67 risk factors and risk factor clusters in 21 regions, 1990–2010: A systematic analysis for the global burden of disease study 2010. Lancet.

[2] Yamazaki S., Shima M., Yoda Y., Oka K., Kurosaka F., Shimizu S., Takahashi H., Nakatani Y., Nishikawa J., Fujiwara K. (2014). Association between PM_2.5_ and primary care visits due to asthma attack in Japan: Relation to beijing’s air pollution episode in January 2013. Environ. Health Prev. Med..

[3] Watanabe M., Yamasaki A., Burioka N., Kurai J., Yoneda K., Yoshida A., Igishi T., Fukuoka Y., Nakamoto M., Takeuchi H. (2011). Correlation between asian dust storms and worsening asthma in western Japan. Allergol. Int..

[4] Watanabe M., Kurai J., Igishi T., Yamasaki A., Burioka N., Takeuchi H., Sako T., Touge H., Nakamoto M., Hasegawa Y. (2012). Influence of Asian desert dust on lower respiratory tract symptoms in patients with asthma over 4 years. Yonago Acta Med..

[5] Pope C.A., Burnett R.T., Thun M.J., Calle E.E., Krewski D., Ito K., Thurston G.D. (2002). Lung cancer, cardiopulmonary mortality, and long-term exposure to fine particulate air pollution. JAMA J. Am. Med. Assoc..

[6] Al-Dhaher F.F., Pope J.E., Ouimet J.M. (2010). Determinants of morbidity and mortality of systemic sclerosis in Canada. Semin. Arthritis Rheum..

[7] Dominici F., Peng R.D., Bell M.L., Pham L., McDermott A., Zeger S.L., Samet J.M. (2006). Fine particulate air pollution and hospital admission for cardiovascular and respiratory diseases. J. Am. Med. Assoc..

[8] Mustafic H., Jabre P., Caussin C., Murad M.H., Escolano S., Tafflet M., Perier M.C., Marijon E., Vernerey D., Empana J.P. (2012). Main air pollutants and myocardial infarction: A systematic review and meta-analysis. JAMA J. Am. Med. Assoc..

[9] Host S., Larrieu S., Pascal L., Blanchard M., Declercq C., Fabre P., Jusot J.F., Chardon B., Le Tertre A., Wagner V. (2008). Short-term associations between fine and coarse particles and hospital admissions for cardiorespiratory diseases in six French cities. Occup. Environ. Med..

[10] Malig B.J., Green S., Basu R., Broadwin R. (2013). Coarse particles and respiratory emergency department visits in California. Am. J. Epidemiol..

[11] Malig B.J., Ostro B.D. (2009). Coarse particles and mortality: Evidence from a multi-city study in California. Occup. Environ. Med..

[12] Chen R., Li Y., Ma Y., Pan G., Zeng G., Xu X., Chen B., Kan H. (2011). Coarse particles and mortality in three Chinese cities: The china air pollution and health effects study (CAPES). Sci. Total Environ..

[13] Peng R.D., Chang H.H., Bell M.L., McDermott A., Zeger S.L., Samet J.M., Dominici F. (2008). Coarse particulate matter air pollution and hospital admissions for cardiovascular and respiratory diseases among medicare patients. JAMA J. Am. Med. Assoc..

[14] Weinmayr G., Romeo E., De Sario M., Weiland S.K., Forastiere F. (2010). Short-term effects of PM_10_ and NO_2_ on respiratory health among children with asthma or asthma-like symptoms: A systematic review and meta-analysis. Environ. Health Perspect..

[15] Schwarze P.E., Ovrevik J., Lag M., Refsnes M., Nafstad P., Hetland R.B., Dybing E. (2006). Particulate matter properties and health effects: Consistency of epidemiological and toxicological studies. Hum. Exp. Toxicol..

[16] Atkinson R.W., Kang S., Anderson H.R., Mills I.C., Walton H.A. (2014). Epidemiological time series studies of PM_2.5_ and daily mortality and hospital admissions: A systematic review and meta-analysis. Thorax.

[17] Watanabe M., Noma H., Kurai J., Sano H., Kitano H., Saito R., Kimura Y., Aiba S., Oshimura M., Shimizu E. (2015). Variation in the effect of particulate matter on pulmonary function in schoolchildren in western Japan and its relation with interleukin-8. Int. J. Environ. Res. Public Health.

[18] Salonen R.O., Halinen A.I., Pennanen A.S., Hirvonen M.R., Sillanpaa M., Hillamo R., Shi T., Borm P., Sandell E., Koskentalo T. (2004). Chemical and *in vitro* toxicologic characterization of wintertime and springtime urban-air particles with an aerodynamic diameter below 10 microm in Helsinki. Scand. J. Work Environ. Health.

[19] Hetland R.B., Cassee F.R., Lag M., Refsnes M., Dybing E., Schwarze P.E. (2005). Cytokine release from alveolar macrophages exposed to ambient particulate matter: Heterogeneity in relation to size, city and season. Part. Fibre Toxicol..

[20] Jalava P.I., Hirvonen M.R., Sillanpaa M., Pennanen A.S., Happo M.S., Hillamo R., Cassee F.R., Gerlofs-Nijland M., Borm P.J., Schins R.P. (2009). Associations of urban air particulate composition with inflammatory and cytotoxic responses in raw 246.7 cell line. Inhal. Toxicol..

[21] Kumar R.K., Shadie A.M., Bucknall M.P., Rutlidge H., Garthwaite L., Herbert C., Halliburton B., Parsons K.S., Wark P.A. (2015). Differential injurious effects of ambient and traffic-derived particulate matter on airway epithelial cells. Respirology.

[22] Zanobetti A., Schwartz J. (2009). The effect of fine and coarse particulate air pollution on mortality: A national analysis. Environ. Health Perspect..

[23] Ichinose T., Yoshida S., Sadakane K., Takano H., Yanagisawa R., Inoue K., Nishikawa M., Mori I., Kawazato H., Yasuda A. (2008). Effects of asian sand dust, arizona sand dust, amorphous silica and aluminum oxide on allergic inflammation in the murine lung. Inhal. Toxicol..

[24] Ichinose T., Yoshida S., Hiyoshi K., Sadakane K., Takano H., Nishikawa M., Mori I., Yanagisawa R., Kawazato H., Yasuda A. (2008). The effects of microbial materials adhered to asian sand dust on allergic lung inflammation. Arch. Environ. Contam. Toxicol..

[25] He M., Ichinose T., Yoshida S., Nishikawa M., Mori I., Yanagisawa R., Takano H., Inoue K., Sun G., Shibamoto T. (2010). Airborne Asian sand dust enhances murine lung eosinophilia. Inhal. Toxicol..

[26] Shibamori M., Ogino K., Kambayashi Y., Ishiyama H. (2006). Intranasal mite allergen induces allergic asthma-like responses in NC/Nga mice. Life Sci..

[27] Cao J. (2012). Pollution status and control strategies of PM_2.5_ in China. J. Earth Environ..

[28] Wang Y., Zhang R., Saravanan R. (2014). Asian pollution climatically modulates mid-latitude cyclones following hierarchical modelling and observational analysis. Nat. Commun..

[29] Hioki T., Nakanishi S., Mukai H., Murano K., Ohara T., Wakamatsu S. (2008). Analysis of long-range transported and local air pollution with trace metal concentration ratio and lead isotope ratio in precipitation. J. Jpn. Soc. Atmos. Environ..

[30] Wuebbles D.J., Lei H., Lin J. (2007). Intercontinental transport of aerosols and photochemical oxidants from Asia and its consequences. Environ. Pollut..

[31] Hioki T., Kimoto T., Hasegawa S., Mukai H., Ohara T., Wakamatsu S. (2009). Analysis of long-lange transported and local air pollution with trace metal concentration ratio in aerosols collected at Matsuyama, Osaka and Tsukuba, Japan. J. Jpn. Soc. Atmos. Environ..

[32] Yamada E., Funoki S., Abe Y., Umemura S., Yamaguchi D., Fuse Y. (2005). Size distribution and characteristics of chemical components in ambient particulate matter. Anal. Sci. Int. J. Jpn. Soc. Anal. Chem..

[33] Kurai J., Watanabe M., Tomita K., Yamasaki H.S., Shimizu E. (2014). Influence of asian dust particles on immune adjuvant effects and airway inflammation in asthma model mice. PLoS ONE.

[34] Becker S., Dailey L.A., Soukup J.M., Grambow S.C., Devlin R.B., Huang Y.C. (2005). Seasonal variations in air pollution particle-induced inflammatory mediator release and oxidative stress. Environ. Health Perspect..

[35] Plummer L.E., Ham W., Kleeman M.J., Wexler A., Pinkerton K.E. (2012). Influence of season and location on pulmonary response to California’s San Joaquin Valley airborne particulate matter. J. Toxicol. Environ. Health Part A.

[36] Farina F., Sancini G., Mantecca P., Gallinotti D., Camatini M., Palestini P. (2011). The acute toxic effects of particulate matter in mouse lung are related to size and season of collection. Toxicol. Lett..

[37] Mirowsky J., Hickey C., Horton L., Blaustein M., Galdanes K., Peltier R.E., Chillrud S., Chen L.C., Ross J., Nadas A. (2013). The effect of particle size, location and season on the toxicity of urban and rural particulate matter. Inhal. Toxicol..

[38] Alexis N., Eldridge M., Reed W., Bromberg P., Peden D.B. (2001). Cd14-dependent airway neutrophil response to inhaled LPS: Role of atopy. J. Allergy Clin. Immunol..

[39] Alexis N.E., Peden D.B. (2006). Inflammatory response of the airway to inhaled endotoxin correlates with body mass index in atopic patients with asthma but not in normal volunteers. J. Allergy Clin. Immunol..

[40] Goldsmith C.A., Hamada K., Ning Y., Qin G., Catalano P., Krishna Murthy G.G., Lawrence J., Kobzik L. (1999). Effects of environmental aerosols on airway hyperresponsiveness in a murine model of asthma. Inhal. Toxicol..

[41] Zosky G.R., Iosifidis T., Perks K., Ditcham W.G., Devadason S.G., Siah W.S., Devine B., Maley F., Cook A. (2014). The concentration of iron in real-world geogenic PM_10_ is associated with increased inflammation and deficits in lung function in mice. PLoS ONE.

[42] Chen R., Zhao Z., Kan H. (2013). Heavy smog and hospital visits in Beijing, China. Am. J. Respir. Crit. Care Med..

[43] Coulibaly S., Minami H., Abe M., Furukawa N., Ono R., Hasei T., Toriba A., Tang N., Hayakawa K., Funasaka K. (2016). Comparison of air pollution in metropolises in China (Beijing) and Japan (Osaka and Nagoya) on the basis of the levels of contaminants and mutagenicity. Biol. Pharm. Bull..

[44] Zhang H., Wang Y., Hu J., Ying Q., Hu X.M. (2015). Relationships between meteorological parameters and criteria air pollutants in three megacities in China. Environ. Res..

[45] Kim K.H., Hong Y.J., Szulejko J.E., Kang C.H., Chambers S., Feng X., Deep A., Kim Y.H. (2016). Airborne iron across major urban centers in South Korea between 1991 and 2012. Sci. Total Environ..

[46] Ishibashi Y., Ohno H., Oh-ishi S., Matsuoka T., Kizaki T., Yoshizumi K. (2008). Characterization of pollen dispersion in the neighborhood of Tokyo, Japan in the spring of 2005 and 2006. Int. J. Environ. Res. Public Health.

[47] Brook R.D., Franklin B., Cascio W., Hong Y., Howard G., Lipsett M., Luepker R., Mittleman M., Samet J., Smith S.C. (2004). Air pollution and cardiovascular disease: A statement for healthcare professionals from the expert panel on population and prevention science of the American heart association. Circulation.

